# Downregulation of AC092894.1 promotes oxaliplatin resistance in colorectal cancer via the USP3/AR/RASGRP3 axis

**DOI:** 10.1186/s12916-023-02826-6

**Published:** 2023-04-03

**Authors:** Zhijian Zheng, Ming Wu, Hongyan Li, Wenxia Xu, Mengxiang Yang, Kailing Pan, Yuqi Ni, Ting Jiang, Hongjuan Zheng, Xiayun Jin, Yanfei Zhang, Linchao Ding, Jianfei Fu

**Affiliations:** 1 Department of Medical Oncology, Affiliated Jinhua Hospital, Zhejiang University School of Medicine, Jinhua, 321000 China; 2 Department of Central Laboratory, Affiliated Jinhua Hospital, Zhejiang University School of Medicine, Jinhua, 321000 China; 3 Department of Clinical Laboratory, Affiliated Jinhua Hospital, Zhejiang University School of Medicine, Jinhua, 321000 China; 4 Key Laboratory of Laboratory Medicine, Ministry of Education, Zhejiang Provincial Key Laboratory of Medical Genetics, School of Laboratory Medicine and Life Sciences, Wenzhou Medical University, Wenzhou, 325035 China; 5 Department of Nuclear Medicine, Affiliated Jinhua Hospital, Zhejiang University School of Medicine, Jinhua, 321000 China; 6 Department of Pathology, Affiliated Jinhua Hospital, Zhejiang University School of Medicine, Jinhua, 321000 China; 7 Department of Scientific Research, Affiliated Jinhua Hospital, Zhejiang University School of Medicine, Jinhua, 321000 China

**Keywords:** AC092894.1, USP3/AR/RASGRP3, Colorectal cancer, Oxaliplatin resistance

## Abstract

**Background:**

Oxaliplatin resistance is a complex process and has been one of the most disadvantageous factors and indeed a confrontation in the procedure of colorectal cancer. Recently, long non-coding RNAs (lncRNAs) have emerged as novel molecules for the treatment of chemoresistance, but the specific molecular mechanisms mediated by them are poorly understood.

**Methods:**

The lncRNAs associated with oxaliplatin resistance were screened by microarray. lncRNA effects on oxaliplatin chemoresistance were then verified by gain- and loss-of-function experiments. Finally, the potential mechanism of AC092894.1 was explored by RNA pull-down, RIP, and Co-IP experiments.

**Results:**

AC092894.1 representation has been demonstrated to be drastically downregulated throughout oxaliplatin-induced drug-resistant CRC cells. In vivo and in vitro experiments revealed that AC092894.1 functions to reverse chemoresistance. Studies on the mechanism suggested that AC092894.1 served as a scaffold molecule that mediated the de-ubiquitination of AR through USP3, thereby increasing the transcription of RASGRP3. Finally, sustained activation of the MAPK signaling pathway induced apoptosis in CRC cells.

**Conclusions:**

In conclusion, this study identified AC092894.1 as a suppressor of CRC chemoresistance and revealed the idea that targeting the AC092894.1/USP3/AR/RASGRP3 signaling axis is a novel option for the treatment of oxaliplatin resistance.

**Supplementary Information:**

The online version contains supplementary material available at 10.1186/s12916-023-02826-6.

## Background

The incidence and mortality rate of colorectal cancer (CRC) are increasing each year. CRC is a common gastrointestinal malignancy with a rising incidence and mortality rate [1]. Currently, oxaliplatin-based chemotherapy is highly effective in locally advanced CRC or metastatic CRC [2, 3]. Oxaliplatin, a third-generation chemotherapeutic agent, works by reacting with DNA in the S-phase to form intra- and inter-strand cross-linked hydrated derivatives. This in turn triggers DNA damage and ultimately apoptosis to achieve therapeutic effects [4, 5]. Notwithstanding, roughly 50% of stage II and III CRC caregivers develop acquired oxaliplatin resistance [6]. Consequently, understanding the molecular processes of oxaliplatin resistance seems to be of the greatest priority.

Long non-coding RNAs (LncRNAs) are translations that exceed 200 nucleic acids in duration and cannot encode proteins [7]. Current findings have shown that lncRNAs can affect cellular processes via multiple pathways, including transcriptions, post-transcriptional modifications, and translations [8, 9]. Additionally, there is growing evidence that lncRNAs interact significant roles in the emergence of drug resistance [10–13]. However, very little research has been done on the molecular mechanisms of lncRNA resistance to oxaliplatin.

In this study, we identified a novel lncRNA, AC092894.1, localized on human chromosome 3 from microarray analysis. Further research showed that in CRC cells that were resistant to drugs, it was markedly downregulated. For mechanistic studies, it was shown that AC092894.1 acted as a scaffold molecule that recruited USP3 to the androgen receptor (AR) and promoted AR transcription. In turn, AR promoted the transcription of RASGRP3, which ultimately activated the MAPK signaling pathway and promoted apoptosis. Our research should lead to the identification of novel therapeutic targets for oxaliplatin-resistant colorectal cancer.

## Methods

### Pathology specimens

Forty tissues from patients with CRC were obtained with their informed consent from the Department of Anorectal Surgery at Affiliated Jinhua Hospital, Zhejiang University School of Medicine (Table S1). The project was approved by the Medical Ethics Committee of Affiliated Jinhua Hospital, Zhejiang University School of Medicine ((2021) Ethics Approval No. (178)). During the process of isolating the tissues, they were flash-frozen in liquid nitrogen; 1/3 of the samples underwent RNA extraction for RT-qPCR validation. The surviving tissue was formalin-fixed and paraffin-embedded. We classified as oxaliplatin-resistant those CRC patients whose disease progressed within 6 months of oxaliplatin administration, and as oxaliplatin-sensitive those who progressed beyond 6 months.

### Culture of cells

LoVo and HCT116 human CRC cells were primarily acquired by the Shanghai Branch of the Chinese Academy of Sciences. In RPMI 1640 medium (GNM31800-5, GENOM) supplemented with 10% fetal bovine serum, LoVo cells were cultured (FBS; 04–001-1ACS; Biological Industries). HCT116 cells were grown in M5A medium (GNM16600-2, GENOM) with 10% FBS supplement. Two very different cells were created in a 37 °C environment with 5% atmospheric CO_2_.

### Creation of the CRC-OxR cell line

By intermittently adding oxaliplatin to the medium containing the parental sensitive cell cultures, LoVo-OxR together with HCT116-OxR cells were created. The concentration of oxaliplatin was gradually increased from 0 to 4 μg/ml for a duration of up to 6 months throughout the process. Oxaliplatin was maintained at 4 μg/ml prior to the experiment in order to maintain cell resistance to oxaliplatin, as previously described [14]. Once the resistant cells are established, their resistance can be stably maintained over 10 generations.

### Reagents, plasmids, and antibodies

From TsingKe Bio, overexpression plasmids AC092894.1, flag-USP3, HA-AR, and their associated control plasmids were bought to Tech. Co. (Beijing, China). The shRNA of AC092894.1 was purchased from Tech. Co. (Beijing, China) and Sequence in Table S3. Additionally, siRNA for USP3 and RASGRP3 ended up being bought from Ruibo Bio. Co. (Guangzhou, China). AR siRNA was purchased from TsingKe Bio. Tech. Co. The RASGRP3 promoter plasmid ended up being bought from Bio. Tech. Co. Antibodies against p38 (ET1702-65), p-p38 (ER2001-52), and FOXP3 (ET1702-12) were purchased from HUABIO. Proteintech was used to acquire the following antibodies: GAPDH (60,004–1-lg), ATF2 (14,834–1-AP), RASGRP3 (13,162–1-AP), α-Tubulin (11,224–1-AP), GATA-2 (11,103–1-AP), RXRα (21,218–1-AP), and USP3 (12,490–1-AP). Cell Signaling Tech. Co. sold us immune responses against C-PARP (Lot No. 5625) and AR (Lot No. 19672).

### Lentiviral infection

As per our previous study [15], 6-well plates were seeded with 293 T cells (10% serum for DMEM). When cell density reached 70–80%, 1.2-μg psPAX.2 (Lot No. 12260, Addgene), 1.2-μg pMD2.G (Lot No. 12259, Addgene), 2-μg plasmid of 293 T, and 50-μL serum-free DMEM with 6 μL of Polyjet were combined together. The cells were cultured in a 6-well plate in a cell incubator for 6–8 h after the addition of the medium, which had been pre-incubated at room temperature for 20 min. After that time, the medium was discarded and DMEM with 10% FBS was added. Once the 48 h were up, the supernatant was centrifuged at 3000 rpm for 20 min before being filtered through a 0.45-μm membrane. Stably transfected cell lines were screened with puromycin after the virus supernatant and medium were added at a 1:1 ratio.

### RT-qPCR (reverse transcription-quantitative PCR)

Trizol (Carlsbad, CA, USA) was used to extract total RNA from cells, and the RNA was reverse-transcribed into cDNA using a Quantscript RT kit as soon as possible after extraction (Takara, Osaka, Japan). Then, the LightCycler® 480II qPCR System was used for RT-qPCR analysis (Roche, Basel, Switzerland). Experiment primers are detailed in Table S2.

### Evaluation by Western blotting

The protein concentrations were measured with a method described kit after cells were lysed in RIPA lysis solution (Beyotime, Shanghai, China). Proteins of equal amounts were run on SDS-PAGE gels and then blotted onto the pore structure. After being blocked with 5% non-fat milk for 10 min at room temperature, the sample was incubated with the primary antibody of interest overnight at 4 °C. The ECL kit (Lot No. RPN5787, GE Healthcare, PA, USA) was used to visualize the amino acids, and the BioRad ChemiDoc XRS + was used to photograph the membranes (CA, USA).

### CCK-8 assay

After 24 h of adhesion, we replaced the complete medium with 5 μg/mL oxaliplatin for 48 h after seeding 1 × 10^4^ LoVo-OxR (AC092894.1), HCT116-OxR (AC092894.1), and control vector cells in 96-well plates containing 200-μL complete medium. Then, we added 10 μL of CCK-8 reagent (Beyotime, Shanghai, China) to each well and incubated them at 37 °C in a shaker for 1 h. The OD values at 450 nm were measured by a microplate reader (Synergy HT ZX-22, Bio-Tek Instruments, VT, USA).

### Colony formation assay

After 24 h of adhesion, complete medium was replaced with 5 μg/mL oxaliplatin for 48 h after seeding 1 × 10^4^ LoVo-OxR(AC092894.1), HCT116-OxR (AC092894.1), and their control vector cells in 96-well plates containing 200 μL complete medium. After that, we added 10 μL of CCK-8 reagent (Beyotime, Shanghai, China) to each well and left them in a 37 °C incubator for 1 h on a rotational shaker. In order to determine the OD at 450 nm, a microplate reader was used (Synergy HT ZX-22, Bio-Tek Instruments, VT, USA).

### Flow cytometry analysis

LoVo-OxR (AC092894.1), HCT116-OxR (AC092894.1), and their control vector cells were seeded into 6-well plates; after 48 h, cells were treated with EDTA-free trypsin (Gibco BRL, Grand Island, NY, USA) for digestion, then washed once with PBS, and incubated with APC and 7-AAD (MultiSciences, Hangzhou, China) for 10 min and analysis was performed by flow cytometry (Beckman Coulter, Fullerton, CA, USA).

### Nude mouse xenograft model

BALB/c nude mice were purchased from Vital River (Beijing, China), and 3–4-week-old nude mice were selected. The mice were divided into 4 groups, that is, control group, AC092894.1 overexpression group, control plus oxaliplatin group, and AC092894.1 overexpression plus oxaliplatin group. 4 × 10^6^ HCT116-vector and HCT116-AC092894.1 cells were seeded subcutaneously on the right ventral side of the mice. About 2 weeks later, oxaliplatin was injected intraperitoneally at a dose of 5 mg/kg/day per mice. After about 1 month, mice were euthanized and tumors were surgically removed, photographed, and weighed. The study for the animals was approved by the Experimental Animal Welfare and Ethics Committee of Affiliated Jinhua Hospital, Zhejiang University School of Medicine (Approval No. AL-JHYY202207).

### Immunohistochemistry

Paraffin-embedded tissue was cut into 4–5-μm sections, de-paraffinized, and hydrated with ethanol and xylene. Antigen retrieval was performed in boiling citrate buffer for 20 min. Tissues were incubated with H_2_O_2_, blocked with 5% BSA for 30 min, and then incubated overnight at 4 °C with the corresponding primary antibody. For IHC staining, antibodies against p-p38 were used.

### The RNA pull-down test

With the help of a kit, we were able to successfully complete an RNA pull-down (BersinBio, Bes5102N, Guangzhou, China). AC092894.1’s biotin probe was developed by Mingrui Technology (Guangzhou, China). The CRC cell lysate was incubated with 2 μg of the probe and magnetic beads and then eluted for protein-MS and Western blotting.

### RIP-qPCR

Experiments with RNA immunoprecipitation (RIP) kits were conducted (BersinBio, bes5101, Guangzhou, China). AC092894.1 was detected by RT-qPCR after being captured by 3 μg of AR (Lot No. 19672), flag (F1804-5MG, sigma, USA), and an IgG control antibody.

### Bioinformatics

The coding potential of AC092894.1 was detected using CPC2.0 (http://cpc2.gao-lab.org/) to determine that it is a non-coding RNA. Predictive analysis of the promoter region of RASGRP3 using the PROMO database(http://alggen.lsi.upc.es/cgi-bin/promo_v3/promo/promoinit.cgi?dirDB=TF 8.3).

### Statistical analysis

Graph generation and data analysis were carried out using GraphPad Prism 7. Experimental data were expressed as mean ± standard deviation. The significance of the differences between the groups was analyzed using the Student’s *t*-test. Significance was based on a threshold of *P* < 0.05.

## Results

### The expression of AC092894.1 is reduced in colorectal cancer (CRC) cells and patient tissues that are resistant to oxaliplatin

**Fig. 1:**
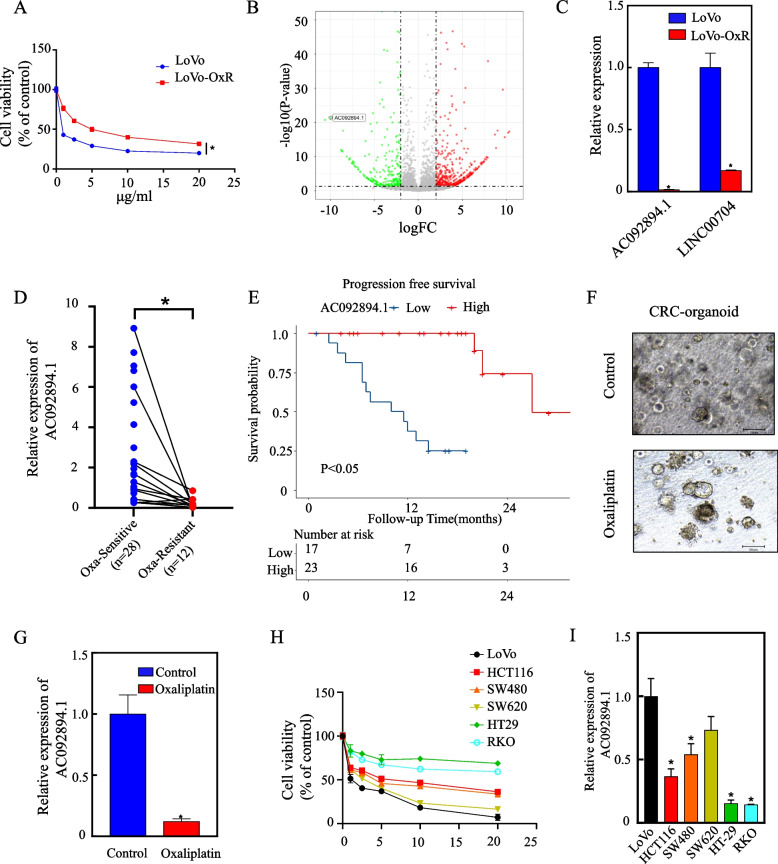
Evidence for AC092894.1 being downregulated in oxaliplatin-resistant CRC cells. **A** LoVo and LoVo-OxR cells were treated with high concentrations of oxaliplatin for 48 h to determine cell viability. **B** Volcano plot showing differentially expressed lncRNAs in LoVo and LoVo-OxR cells. **C** RT-qPCR assays for the expression of the top two most downregulated lncRNAs. **D** Expression of AC092894.1 in our cohort of CRC patients sensitive (*n* = 28) and resistant (*n* = 22) to oxaliplatin treatment. **E** Progression-free survival was analyzed using survival curves. **F**, **G** Microscopic photographs of organs and RT-qPCR detection of AC092894.1 expression in organoids treated with 5 μg/ml oxaliplatin for 72 h. **H** After treatment with the indicated concentrations of oxaliplatin for 48 h, the cell viability of different CRC cells was detected by CCK-8. **I** RT-qPCR assay to detect the expression of AC092894.1 in different CRC cells

We first constructed oxaliplatin-resistant CRC cells LoVo (LoVo-OxR) by continuous drug administration for up to 6 months in order to study the role of lncRNAs in oxaliplatin resistance in CRC. Cell viability assays were used to compare the sensitivity of LoVo-OxR and its parental cells to oxaliplatin, and to calculate the half-maximal inhibitory concentration (IC50) (Fig. 1A). We performed whole-genome sequencing analysis of lncRNAs for LoVo and LoVo-OxR, and the volcano plots show the distribution of differential genes (Fig. 1B). Further, the two most significantly downregulated lncRNAs were screened in the sequencing results. By RT-qPCR, AC092894.1 was confirmed to be the most significantly downregulated (Fig. 1C). Significant downregulation of AC092894.1 was observed in clinical samples of oxaliplatin-resistant CRC, indicating that the AC092894.1 gene may be linked to oxaliplatin resistance in patients (Fig. 1D). In 40 CRC patients, patients with high AC092894.1 expression were observed to have a favorable prognosis (Fig. 1E). Organoids are models with excellent genetic and phenotypic reproducibility [16]. Using the CRC organoid constructed by our team, the expression of AC092894.1 was significantly downregulated by oxaliplatin (Fig. 1F, G). We employed six CRC cell lines exposed to increasing concentrations of oxaliplatin and noticed that HT-29 and RKO were naturally resistant compared with other CRC cell lines, whereas LoVo showed the highest degree of sensitivity (Fig. 1H). Moreover, we adopted RT-qPCR to detect that the expression of AC092894.1 decreased with increasing drug resistance (Fig. 1I). To find out if AC092894.1 was a coding RNA, we used the coding potential calculator CPC2.0. According to the investigation, we have proved that when compared to other genes (GAPDH, ACTB, and ACTA1) and typical non-coding RNAs (Malat1 and Hotair), AC092894.1 had a significantly lower coding potential (Additional file 1: Fig. S1A) [17]. We identified AC092894.1 from chromosome 3 as an intergenic lncRNA consisting of two exons and one intron (Additional file 1: Fig. S1B).

### Increased oxaliplatin sensitivity in CRC cells is associated with ectopic overexpression of AC092894.1

**Fig. 2:**
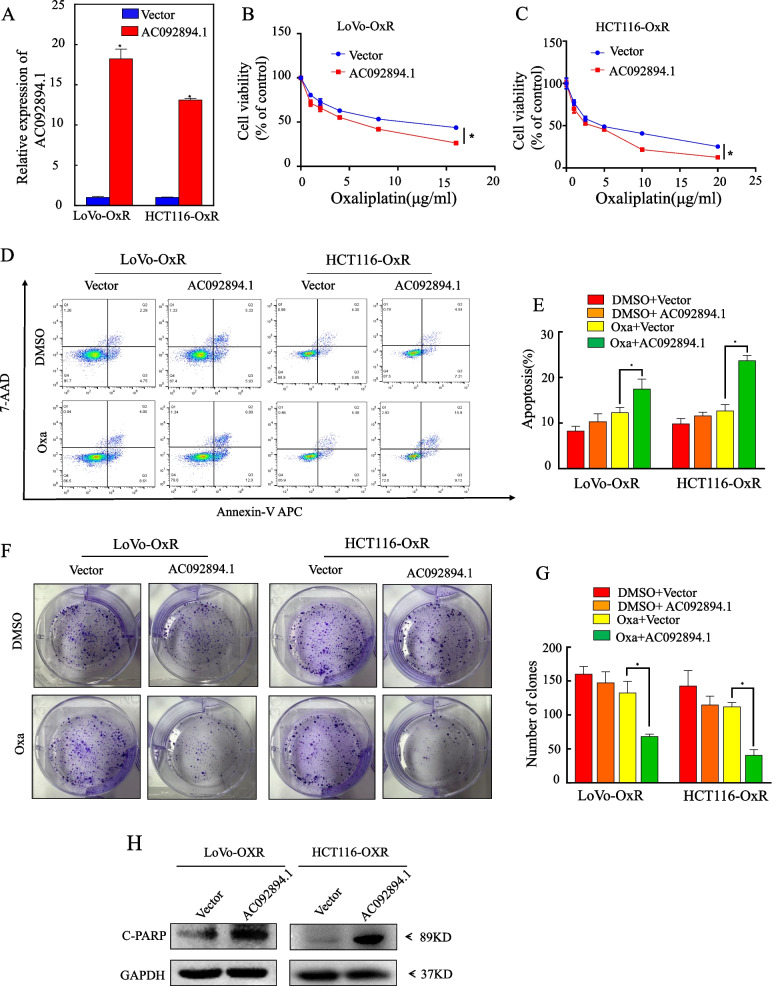
Ectopic overexpression of AC092894.1 promotes chemosensitivity of CRC cells to oxaliplatin. **A** RT-qPCR assay for stable overexpression of AC09894.1. **B**, **C** Overexpression of AC092894.1 on cells detected by CCK-8 after treatment with the indicated concentrations of oxaliplatin for 48 h. **D** The effect of AC092894.1 overexpression on apoptosis detected by flow cytometry after 5 μg/ml oxaliplatin treatment for 48 h. **E** Statistical analysis of apoptosis rates. **F** The influence on colony formation by overexpression of AC092894.1 was examined after treatment with oxaliplatin (5 μg/ml) for 48 h. **G** Counting and statistical analysis of the colony numbers. **I** Western blotting to detect the expression of C-PARP in cell lysates with GAPDH as a control

Oxaliplatin-induced chemosensitivity in colorectal cancer cells is facilitated by ectopic overexpression of AC092894.1. Cell viability assays were used to compare the sensitivity of HCT116-OxR and its parental cells to oxaliplatin and to calculate the half-maximal inhibitory concentration (IC50) (Additional file 1: Fig. S2A). Furthermore, an overexpression plasmid of AC092894.1 was constructed and transfected into LoVo-OxR and HCT116-OxR cells, respectively. Testing its overexpression efficiency revealed that it could be stably expressed (Fig. 2A). Cell viability assays showed that the effect of oxaliplatin on drug-resistant CRC cells that was promoted by overexpression of AC092894.1 increased with increasing concentrations of oxaliplatin (Fig. 2B, C). In way of comparison, knockdown of AC092894.1 in CRC parental cells enhanced cellular tolerance to oxaliplatin (Additional file 1: Fig. S2B-2D). We found an interesting phenomenon in the apoptosis assay that overexpression of AC092894.1 promoted the chemosensitivity of drug-resistant CRC cells only after exposure to oxaliplatin. In contrast, overexpression of AC092894.1 in resistant CRC cells exposed to the control solvent did not promote chemosensitivity in resistant CRC cells (Fig. 2D, E). In parental CRC cells, knockdown of AC092894.1 decreased the rate of apoptosis after exposure to oxaliplatin (Additional file 1: Fig. S2E&2F). We then found the same pattern in colony formation experiments, where overexpression of AC092894.1 significantly promoted chemosensitivity only in oxaliplatin-resistant CRC cells exposed to oxaliplatin (Fig. 2F, G). In parental CRC cells, knockdown of AC092894.1 increased the number of oxaliplatin-exposed cell clones (Additional file 1: Fig. S2G&2H). Western blotting analysis showed that overexpression of AC092894.1 could significantly promote high expression of apoptosis marker C-PARP after oxaliplatin treatment (Fig. 2H). Consequently, we attempted to detect multidrug resistance in LoVo-OxR and HCT116-OxR cells and observed that AC092894.1 likewise reversed resistance to 5-Fu and irinotecan(Additional file 1: Fig. S2I-L).

### CRC cell sensitivity to oxaliplatin is increased by RASGRP3, a downstream effector molecule of AC092894.1

**Fig. 3:**
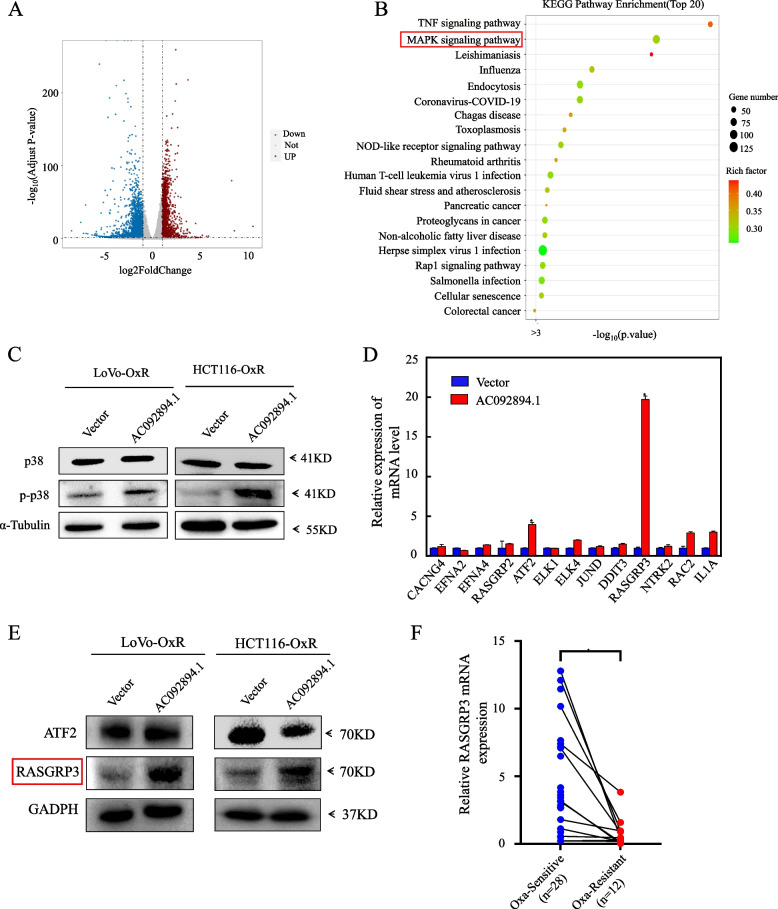
RAGRP3 is a downstream effector molecule of AC092894.1. **A** Volcano plot of differentially expressed genes obtained by RNA-seq. **B** KEGG pathway analysis for AC092894.1. The vertical axis represents the pathway. The horizontal axis represents enrichment factors. The size of the dots indicates the number of differentially expressed genes in the corresponding pathway (gene count). The color of the dots indicates the specific value of *P*-value. **C** Western blotting to detect the expression of p38 and p-p38 in cell lysates, with α-Tubulin as a control. **D** RT-qPCR for expression of MAPK signaling pathway-related molecules in LoVo-OxR after overexpression of AC092894.1. **E** Western blotting to detect the expression of ATF2 and RASGRP3 in cell lysates, with GAPDH was as a control. **F** Expression of AC092894.1 in our cohort of CRC patients sensitive (*n* = 28) and resistant (*n* = 22) to oxaliplatin treatment

We utilized RNA-seq analysis of overexpressed CRC cells and their control vector cells to further investigate the molecular mechanism by which AC092894.1 promotes chemoresistance in CRC cells. To further explore the molecular mechanism by which 894 promotes chemoresistance in CRC cells, we analyzed stably expressed cells of AC092894.1 and their control vector cells by RNA-seq. The volcano plot shows the differentially expressed genes (Fig. 3A). The KEGG enrichment analysis revealed that the majority of the differentially expressed genes after AC092894.1 overexpression were involved in the TNF signaling pathway and MAPK signaling pathway (Fig. 3B). And we detected TNF-α by western blot and noticed that AC092894.1 may not function through the TNF signaling pathway(Additional file 1: Fig. S3A). Oxaliplatin sensitivity in CRC has been shown to be enhanced by the activation of the mitogen-activated protein kinase signaling pathway [18–21]. Our results also revealed that overexpression of AC092894.1 activated the MAPK signaling pathway in CRC-resistant cells (Fig. 3C). Based on these results, AC092894.1 may be involved in the development of oxaliplatin resistance as part of the MAPK signaling pathway. In order to investigate how AC092894.1 is involved in regulating the MAPK signaling pathway, we screened the RNA-seq database for key genes differentially expressed in the MAPK signaling pathway between AC092894.1 overexpression and control (Table S4). Further, these genes were validated by real-time quantitative PCR. The results revealed that RASGRP3 was significantly upregulated at the level of mRNA after AC092894.1 overexpression. Validation in LoVo-OxR and HCT116-OxR also revealed a significant upregulation of RASGRP3 protein levels (Fig. 3D, E; Fig. S3B). It is hypothesized that RASGRP3 may be a downstream effector molecule of AC092894.1. Using quantitative real-time PCR, we discovered that RASGRP3 was significantly downregulated in oxaliplatin-resistant CRC patients (Fig. 3F).

### AC092894.1 mediates the chemoresistance of CRC cells by regulating RASGRP3 expression at the transcriptional level

**Fig. 4:**
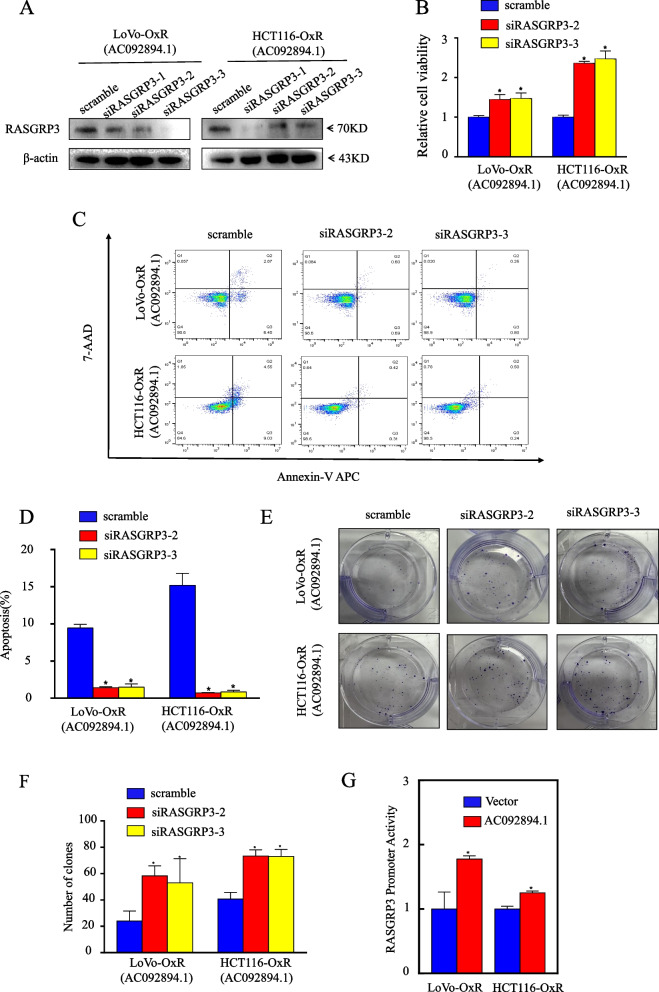
AC092894.1 requires targeting of RASGRP3 to mediate chemosensitivity. **A** Western blotting was performed to detect RASGRP3 expression in cell lysates after silencing RASGRP3 with siRNA in LoVo-OxR (AC092894.1) and HCT116-OxR (AC092894.1) cells. **B** CCK-8 assay of cell viability after oxaliplatin (5 μg /ml) treatment for 48 h. **C** The effect of RASGRP3 knockdown on apoptosis was detected by flow cytometry after oxaliplatin (5 μg /ml) treatment for 48 h. **D** Statistical analysis of apoptosis rates. **E** The effect of RASGRP3 knockdown on colony formation after 48-h treatment oxaliplatin (5 μg/ml). **F** Counting and statistical analysis of the colony numbers. **G** Comparison of RASGRP3 promoter activity of AC092894.1 overexpression cells with vector control cells. **F** The influence on colony formation by overexpression of AC092894.1 was examined after treatment with oxaliplatin (5 μg/ml) for 48 h. **G** Counting and statistical analysis of the colony numbers. **I** Western blotting to detect the expression of C-PARP in cell lysates with GAPDH as a control

To determine whether RASGRP3 is essential for AC092894.1 to mediate oxaliplatin resistance in CRC cells, we knocked down RASGRP3 in LoVo-OxR (AC092894.1) and HCT116-OxR (AC092894.1) cells, respectively (Fig. 4A). The results of cell viability, apoptosis, and colony formation indicated that knockdown of RASGRP3 restored oxaliplatin resistance in drug-resistant CRC cells (Fig. 4B–F). The knockdown of RASGRP3 was also associated with a significant decrease in p-p38 expression, indicating that the MAPK signaling pathway was inhibited (Additional file 1: Fig. S4). Having known that AC092894.1 affects which is the manifestation of RASGRP3 the messenger RNA stage, to investigate this hypothesis, we aimed to determine if AC092894.1 has any effect on RASGRP3 transcriptional activity. Therefore, we constructed the promoter sequence of RASGRP3 into the PGL3-basic vector. It was found that overexpression of AC092894.1 promoted the promoter activity of RASGRP3 via a dual-luciferase reporter gene (Fig. 4G). This result suggested that the altered expression of RASGRP3 was caused by changes at the transcriptional level.

### AR as a transcription factor promotes the transcription of RASGRP3 and mediates chemoresistance

**Fig. 5:**
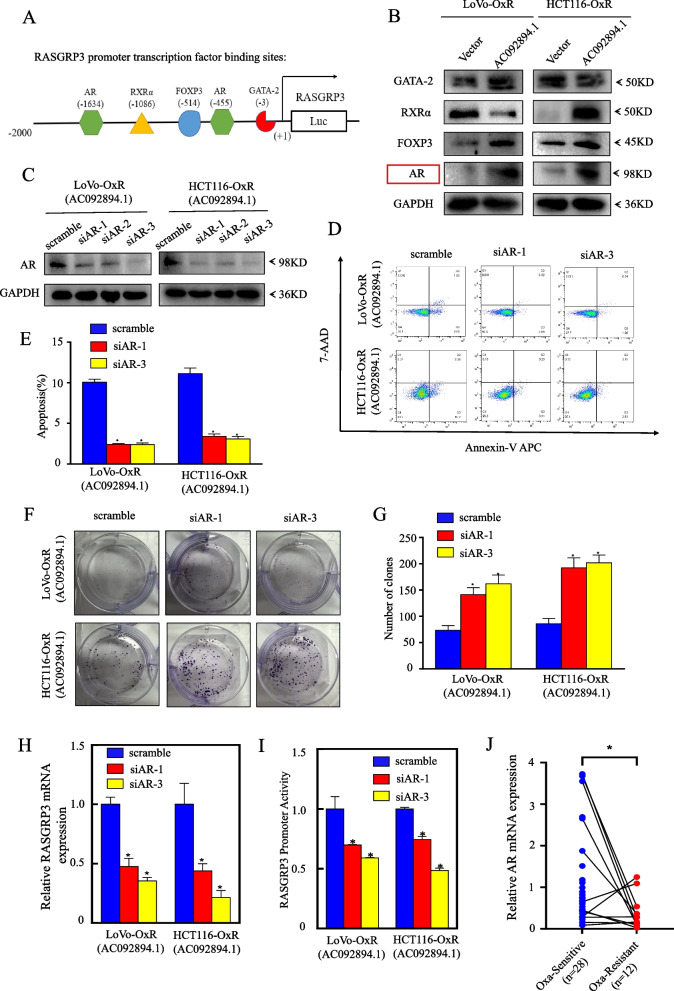
AR promotes RASGRP3 transcription to mediate chemosensitivity. **A** Prediction of potential transcription factors in the RASGRP3 promoter region. **B** Expression of GATA-2, RXRα, FOXP3, and AR was assessed by Western blotting. **C** Western blotting to detect RASGRP3 expression in cell lysates after silencing AR with siRNA in LoVo-OxR (AC092894.1) and HCT116-OxR (AC092894.1) cells. **D** The effect of AR knockdown on apoptosis was examined by flow cytometry after treatment with oxaliplatin (5 μg/ml) for 48 h. **E** Statistical analysis of apoptosis rates. **F** The effect of AR knockdown on colony formation after 48-h treatment with oxaliplatin (5 μg/ml). **G** Counting and statistical analysis of the colony numbers. **H** RT-qPCR assays for RASGRP3 mRNA expression in LoVo-OxR (AC092894.1) and HCT116-OxR (AC092894.1) cells and their corresponding control vector cells. **I** Comparison of RASGRP3 promoter activity in AR knockdown cells with vector control cells. **J** Expression of AR in our cohort of CRC patients sensitive (*n* = 28) and resistant (*n* = 22) to oxaliplatin treatment

We have been applying the PROMO database to predict the RASGRP3 due to having so that we could investigate the transcriptional changes that had occurred. It was found that GATA-2, RXRα, FOXP3, and AR could be their potential transcription factors (Fig. 5A). Further analysis showed that only AR was upregulated in both LoVo-OxR and HCT116-OxR cells after AC092894.1 overexpression (Fig. 5B). It was suggested that AR may be a key factor affecting RASGRP3 transcription. To explore whether AR also mediates chemoresistance, we knocked down AR in LoVo-OxR (AC092894.1) and HCT116-OxR (AC092894.1) cells, respectively (Fig. 5C). As shown in Fig. 5D–G, knockdown of AR enhanced oxaliplatin resistance in drug-resistant CRC cells. The knockdown of AR was further found to restore RASGRP3 mRNA levels and promoter activity (Fig. 5H, I). Real-time quantitative PCR revealed that in oxaliplatin-resistant CRC patients, AR expression was significantly reduced (Fig. 5J).

### AC092894.1 as a scaffold molecule recruits USP3 to promote AR de-ubiquitination

**Fig. 6:**
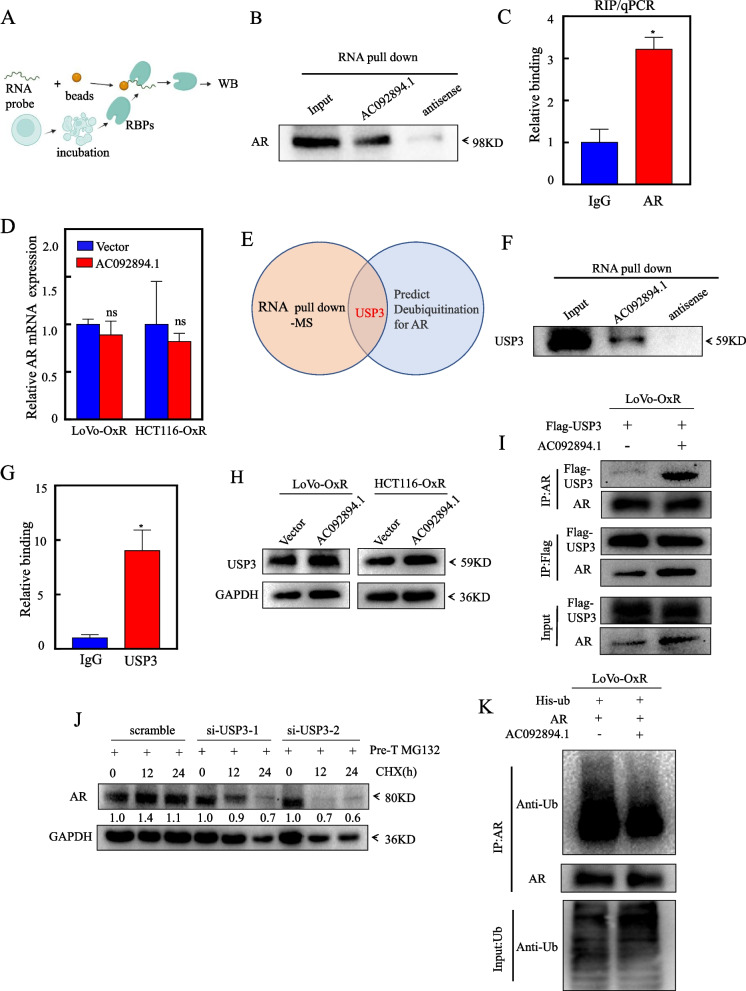
AC092894.1 promotes de-ubiquitination of AR by recruitment of USP3. **A** Schematic diagram of RNA pull-down experiment. **B** Western blotting to detect AR expression after RNA pull down in LoVo-OxR cells. **C** RT-qPCR detection of AC092894.1 expression after RIP experiments. **D** RT-qPCR detection of AR mRNA expression in LoVo-OxR (AC092894.1), HCT116-OxR (AC092894.1), and their corresponding control vector cells. **E** Venn diagrams represent potential AR de-ubiquitinated proteins screened in the RNA-pull down-MS and Ubibrowser database. **F** Western blotting to detect the expression of USP3 in LoVo-OxR cells after RNA pull down. **G** RT-qPCR to detect the expression of AC092894.1 after RIP experiments. **H** Western blotting to assess USP3 expression. **I** The interaction of USP3 and AR was analyzed by co-immunoprecipitation after transfection of Flag-USP3 into LoVo-OxR (AC098894.1) and its control vector cells. **J** For USP3 knockdown LoVo-OxR cells and control vector cells, degradation of AR was detected by Western blotting after treatment with MG132 for 8 h followed by treatment with CHX for the indicated times. **K** His-ub and AR plasmids were co-transfected into LoVo-OxR (AC098894.1) and its control vector cells, and the AR de-ubiquitination was analyzed by immunoblotting with ub antibody

To explore the relationship between AC092894.1 and AR, we hypothesized that AC092894.1 regulates AR by directly combining with it. We used the biotinylated AC092894.1 probe and its antisense probe to perform RNA knockdown, and then Western blotting analysis; see Fig. 6A for the schematic diagram. The results showed that AC092894.1 can capture the characteristics of AR, but the antisense probe is not enriched AR (Fig. 6B). Subsequently, we found through the RIP experiment that compared with IgG, AR antibody can specifically capture AC092894.1 (Fig. 6C). These results demonstrated that AC092894.1 can interact with AR. To explore whether AC092894.1 could affect AR expression at the mRNA level, we detected AR expression in LoVo-OxR and HCT116-OxR cells by RT-qPCR and found that AC092894.1 could not regulate AR at the mRNA level (Fig. 6D). Therefore, AC092894.1 may affect the expression of AR by protein level or other ways. LncRNAs are often reported to participate in signal transduction as guides, decoys, or scaffolds [22]. For this reason, we ventured to speculate whether AC092894.1 functions as a scaffold molecule. Next, we carried out mass spectrometry (MS) analysis of RNA pull-down protein. The intersection of the mass spectrometry and de-ubiquitinase prediction database, Ubibrowser, showed that USP3 may be a potential key molecule (Fig. 6E). Through RNA pull-down experiments, we found that AC092894.1 could capture USP3, but its antisense probe could not, which was consistent with the RNA pull-down-MS (Fig. 6F). Next, we found that AC092894.1 was significantly more enriched on the USP3 antibody than the IgG antibody by RIP assay. This further demonstrated the binding of AC092894.1 to USP3 (Fig. 6G). Moreover, we found that USP3 expression did not change significantly in LoVo-OxR and HCT116-OxR cells after AC092894.1 overexpression, which reinforced our suspicion (Fig. 6H). We determined that USP3 exhibited substantial downregulation in oxaliplatin-resistant CRC patients (Additional file 1: Fig. S5A). We succeed to knock down USP3 (Additional file 1: Fig. S5B) and then found that overexpression of AC092894.1 promoted USP3 interaction with AR by co-immunoprecipitation (Fig. 6I). Experiments found that the knockdown of USP3 promoted the protein degradation of AR (Fig. 6J). Further experiments revealed that overexpression of AC092894.1 promoted the de-ubiquitination of AR (Fig. 6K). These results suggested that AC09894.1 can act as a scaffold molecule that recruits USP3 to AR, promoting its de-ubiquitination and thus increasing the protein level of AR.

### AC092894.1 ectopic overexpression increases oxaliplatin sensitivity in vivo

**Fig. 7:**
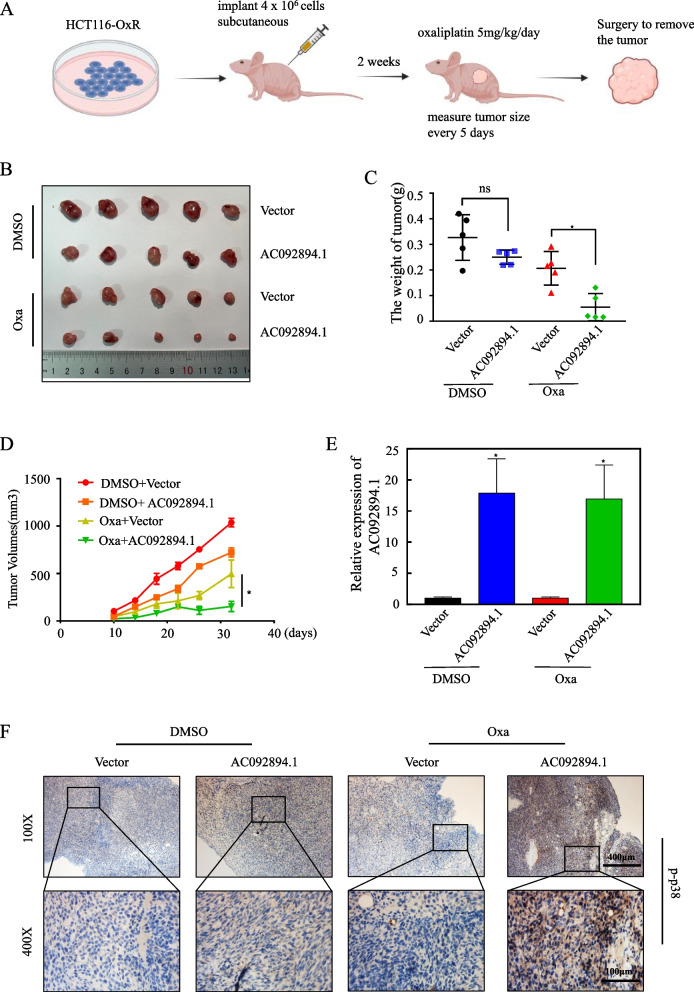
AC098894.1 promotes oxaliplatin sensitization of drug-resistant CRC cells in vivo. **A** Nude mice were subcutaneously xenografted with HCT116-OxR cells (4 × 10^6^ cells) and injected intraperitoneally with oxaliplatin (5 mg/kg/day per mouse) or DMSO every 5 days. **B** Tumor weighing analysis. **C** Tumor growth curves for each group. Data were subjected to Student’s *t*-test. **D** RT-qPCR assays for the expression of AC092894.1 in each group of tumors. **E** Representative IHC images showing p-p38 expression in tumors from each group of mice

**Fig. 8:**
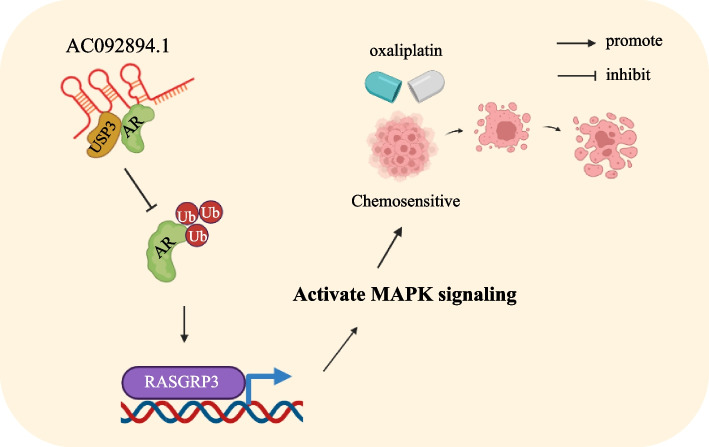
A proposed schematic model of AC092894.1 regulating oxaliplatin resistance in CRC

In order to study the modulatory effect of AC092894.1 on CRC chemoresistance in vivo, we injected HCT116-OxR cells subcutaneously into nude mice. After subcutaneous tumor formation, oxaliplatin (ip, 5 mg/kg per day) was injected intraperitoneally into the mice. The tumor size was measured every 5 days while subjects received either DMSO (0.1 mL/kg) or ethanol (0.1 mL/kg) every 3 days (Fig. 7A). Overexpressing AC092894.1 increased CRC cells’ sensitivity to oxaliplatin, resulting in less dense and smaller tumors (Fig. 7B–D). For mouse tumors, RT-qPCR was used for verification (Fig. 7E). Immunohistochemical experiments indicated that the expression of p-p38 and RASGRP3 increased after AC092894.1 was overexpressed (Fig. 7F, Additional file 1: Fig. S6). In a nutshell, these findings suggest that AC092894.1 may be an effective therapeutic target for overcoming oxaliplatin resistance in CRC patients (Fig. 8).

## Discussion

It is drug resistance that therefore largely limits the treatment strategy for these patients [23, 24]. Patients with advanced or metastatic CRC who are oxaliplatin-resistant tend to have a poor prognosis. This makes it particularly important to investigate and establish an effective oxaliplatin reversal strategy. In the past few years, studies into the role of lncRNA in oxaliplatin opposition in CRC have continued [25]. High levels of Linc00152 and MALAT1, for instance, have been linked to oxaliplatin resistance in colorectal cancer [26, 27]. Oxaliplatin opposition has been linked, among other things, to the overexpression of the genes Linc00152 and MALAT1.

More and more evidence showed that lncRNAs play multiple functions in CRC oxaliplatin resistance [28]. Therefore, it is very reasonable to believe that becoming lncRNAs can become a new diagnostic, prognostic, and therapeutic target for CRC. LncRNAs often function as molecular scaffolds. In addition, the combination of lncRNAs with transcription factors to regulate the expression of downstream genes is the main feedback regulation mode [29, 30]. For example, HNF1A-AS1, as the lncRNA, upregulated OTX1 by binding to the transcription factor PBX3 [31]. Lin et al. [22] found that lncRNA BDNF-AS can promote ubiquitination of RNH1 by recruiting TRIM21. At present, targeting ubiquitinated proteins is a new field of cancer therapy. In this process, molecular glue was like an adhesive, which firmly fixed E3 ubiquitin ligase and target protein together [32]. In our study, AC092894.1 acted as molecular glue, binding with the ubiquitination molecule USP3 and transcription factor AR to induce the ubiquitination of AR.

It has been shown that RasGRP3 can activate both H-Ras and R-Ras, making it a member of the RasGRP family [33]. Ras Guanyl Nucleotide-releasing Peptide (RasGRP) was discovered by Ebinu et al. in 1998, and it functions as a RasGEF [34]. Several studies found that alterations in RASGRP3 expression were linked to breast cancer cell survival and resistance to chemotherapeutic agents [35]. As discovered by Chen et al. [36], GNAQ mutations in uveal melanoma activate the MAPK signaling pathway, and RASGRP3 is the mediator of this activation. In our study, the molecular scaffold AC092894.1 directed USP and AR to regulate RASGRP3. Interestingly, we discovered that RASGRP3 did not regulate the latter after its inhibition (Additional file 1: Fig. S6A-C).

In conclusion, we identified a novel lncRNA, AC092894.1 that was significantly downregulated in oxaliplatin-resistant CRC. Mechanistic studies showed that AC092894.1 can promote the de-ubiquitination of AR by recruitment of USP3. Enabling increased stability of AR can further promote transcription of RASGRP3, which leads to activation of the mitogen-activated protein kinase signaling pathway and finally to chemosensitivity in CRC cells. Our findings demonstrate that AC092894.1 is critical for oxaliplatin opposition and recognize potential therapeutic use of AC092894.1 and its downstream effects in CRC.

## Supplementary Information

**Additional file 1**: **Fig. S1.** The coding potential of AC092894.1 and the gene location. (A)Coding potential score of AC092894.1measured based on CPC2.0. (B) Genomic structure of AC092894.1. **Fig. S2.** Knockdown of AC092894.1 inhibits the sensitivity of CRC cells to oxaliplatin. (A) Cell viability of HCT116 and HCT116-OxR cells treated with different concentrations of oxaliplatin for 48 h. (B) Knockdown efficiency of AC092894.1 detected by RT-qPCR. (C-H) The effect of oxaliplatin treatment after 48h was detected by CCK-8, colon formation, and apoptosis after knockdown of AC092894.1. (I) At the indicated concentrations, CCK-8 assayed the sensitivity of HCT116-OxR to 5-Fu. (J) At the indicated concentrations, CCK-8 assayed the sensitivity of HCT116-OxR to Irinotecan. (K) Overexpression of AC092894.1 on cells detected by CCK-8 after treatment with the indicated concentrations of 5-Fu for 48 h. (L) Overexpression of AC092894.1 on cells detected by CCK-8 after treatment with the indicated concentrations of Irinotecan for 48 h. **Fig. S3.** AC092894.1 does not promote chemo-sensitivity in CRC cells via the TNF signaling pathway.(A) Western blotting to assess TNF-α expression. (B)RT-qPCR assay of RASGRP3 expression in HCT116-OxR after overexpression of AC092894.1. **Fig. S4.** Effect of knockdown of RASGRP3 on p-p38 expression.Expression of p-p38 in cell lysates detected by western blotting; α-Tubulin was used as a control. **Fig. S5.** Expression of USP3 in patient tissues and detection of knockdown efficiency in cells. (A) Expression of USP3 in our cohort of CRC patients sensitive (n = 28) and resistant (n=22) to oxaliplatin treatment. (B) Western blotting to detect USP3 expression in cell lysates after silencing USP3 by siRNA in LoVo-OxR (AC092894.1) cells. **Fig. S6.** Effect of overexpression of AC092894.1 on RASGRP3 expression in mouse tumors. Representative IHC images showing RASGRP3 expression in tumors from each group of mice. **Fig. S7.** Effect of knockdown of RASGRP3 on AC092894.1/USP/AR.(A)RT-qPCR to detect the expression of AC092894.1.(B) Western blotting to assess AR expression. (C) Western blotting to assess USP3 expression.

**Additional file 2:** **Table S1.** The clinical information of CRC patients. **Table S2.** The list of qPCR primers in this study. **Table S3.** Sequence of shRNA. **Table S4.** List of genes with >2-fold differential expression in the MAPK signalling pathway from RNA-seq results.

## Data Availability

Raw transcriptome sequencing data (PRJNA880413) for lncRNAs in LoVo and LoVo-OxR have been uploaded to the NCBI Sequence Read Archive (SRA). RNA-seq raw transcriptome sequencing data on LoVo-OxR-AC092894.1 and control vector cells (PRJNA940036) have been uploaded to the NCBI SRA.

## Abbreviations

AR: Androgen receptor
CRC: Colorectal cancer
LncRNA: Long non-coding RNAs
MS: Mass spectrometry
RasGRP: Ras guanyl nucleotide-releasing peptide
RIP: RNA immunoprecipitation
SRA: Sequence read archive
