# Supplementary material for: Downregulation of AC092894.1 promotes oxaliplatin resistance in colorectal cancer via the USP3/AR/RASGRP3 axis
Source: BMC Med. 2023 Apr 3;21:132. doi: 10.1186/s12916-023-02826-6 (PMC10071743; doi:10.1186/s12916-023-02826-6)
Supplement: Supplementary file 1 — Additional file 1: Fig. S1. The coding potential of AC092894.1 and the gene location. (A)Coding potential score of AC092894.1measured based on CPC2.0. (B) Genomic structure of AC092894.1. Fig. S2. Knockdown of AC092894.1 inhibits the sensitivity of CRC cells to oxaliplatin. (A) Cell viability of HCT116 and HCT116-OxR cells treated with different concentrations of oxaliplatin for 48 h. (B) Knockdown efficiency of AC092894.1 detected by RT-qPCR. (C-H) The effect of oxaliplatin treatment after 48h was detected by CCK-8, colon formation, and apoptosis after knockdown of AC092894.1. (I) At the indicated concentrations, CCK-8 assayed the sensitivity of HCT116-OxR to 5-Fu. (J) At the indicated concentrations, CCK-8 assayed the sensitivity of HCT116-OxR to Irinotecan. (K) Overexpression of AC092894.1 on cells detected by CCK-8 after treatment with the indicated concentrations of 5-Fu for 48 h. (L) Overexpression of AC092894.1 on cells detected by CCK-8 after treatment with the indicated concentrations of Irinotecan for 48 h. Fig. S3. AC092894.1 does not promote chemo-sensitivity in CRC cells via the TNF signaling pathway.(A) Western blotting to assess TNF-α expression. (B)RT-qPCR assay of RASGRP3 expression in HCT116-OxR after overexpression of AC092894.1. Fig. S4. Effect of knockdown of RASGRP3 on p-p38 expression.Expression of p-p38 in cell lysates detected by western blotting; α-Tubulin was used as a control. Fig. S5. Expression of USP3 in patient tissues and detection of knockdown efficiency in cells. (A) Expression of USP3 in our cohort of CRC patients sensitive (n = 28) and resistant (n=22) to oxaliplatin treatment. (B) Western blotting to detect USP3 expression in cell lysates after silencing USP3 by siRNA in LoVo-OxR (AC092894.1) cells. Fig. S6. Effect of overexpression of AC092894.1 on RASGRP3 expression in mouse tumors. Representative IHC images showing RASGRP3 expression in tumors from each group of mice. Fig. S7. Effect of knockdown of RASGRP3 on AC092894.1/USP/ [file 12916_2023_2826_MOESM1_ESM.pptx]

## Slide 1
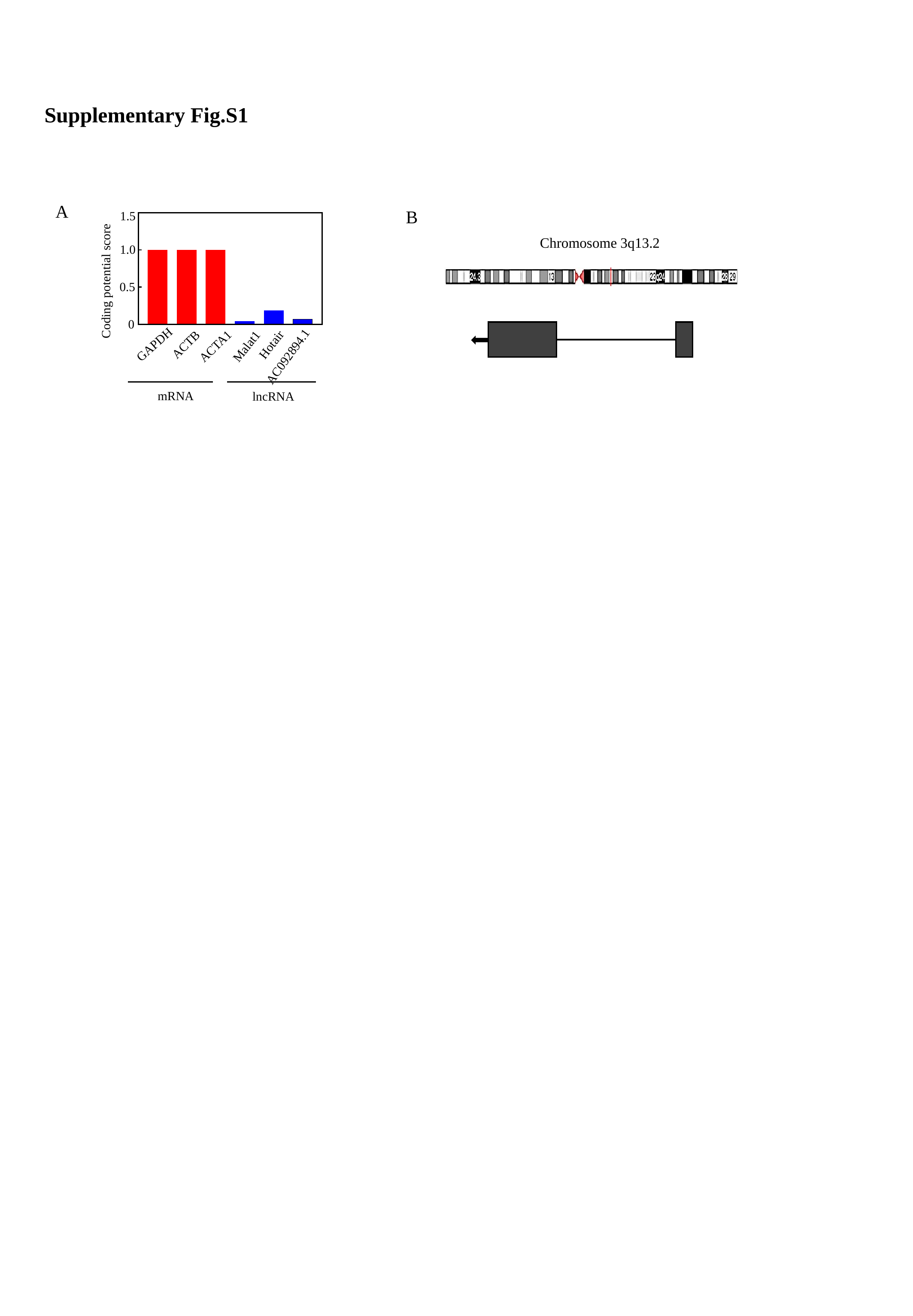

Supplementary Fig.S1
A
1.5
Coding potential score
1.0
0.5
0
GAPDH
ACTB
Hotair
ACTA1
Malat1
AC092894.1
mRNA
lncRNA
B
Chromosome 3q13.2

## Slide 2
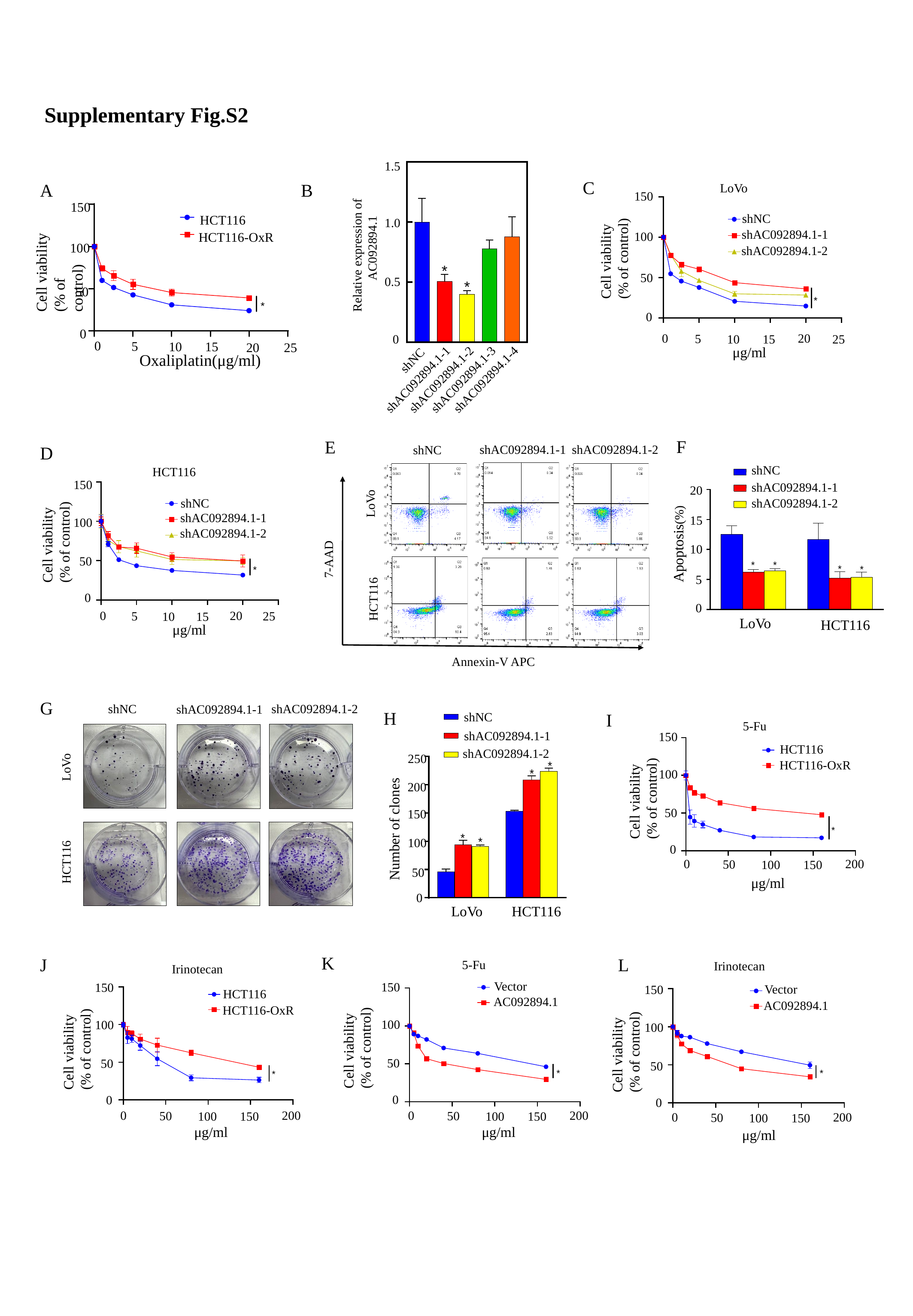

Supplementary Fig.S2
1.5
1.0
0.5
0
Relative expression of
 AC092894.1
shNC
shAC092894.1-2
shAC092894.1-3
shAC092894.1-4
shAC092894.1-1
C
A
B
LoVo
150
100
50
0
shNC
shAC092894.1-1
Cell viability
(% of control)
shAC092894.1-2
20
0
5
25
10
15
μg/ml
150
100
50
0
HCT116
HCT116-OxR
Cell viability
(% of control)
0
5
10
15
20
25
Oxaliplatin(μg/ml)
F
E
D
shAC092894.1-2
shAC092894.1-1
shNC
7-AAD
Annexin-V APC
 LoVo
HCT116
shNC
shAC092894.1-1
20
shAC092894.1-2
15
Apoptosis(%)
10
5
0
LoVo
HCT116
HCT116
150
100
50
0
shNC
shAC092894.1-1
Cell viability
(% of control)
shAC092894.1-2
20
0
5
25
10
15
μg/ml
G
shAC092894.1-2
shNC
shAC092894.1-1
 LoVo
HCT116
shNC
shAC092894.1-1
shAC092894.1-2
250
200
150
100
50
0
Number of clones
LoVo
HCT116
H
I
5-Fu
150
100
50
0
HCT116
HCT116-OxR
Cell viability
(% of control)
200
0
50
100
150
μg/ml
K
L
J
5-Fu
Vector
150
100
50
0
AC092894.1
Cell viability
(% of control)
200
0
50
100
150
μg/ml
Irinotecan
Vector
150
100
50
0
AC092894.1
Cell viability
(% of control)
200
0
50
100
150
μg/ml
Irinotecan
150
100
50
0
HCT116
HCT116-OxR
Cell viability
(% of control)
200
0
50
100
150
μg/ml

## Slide 3
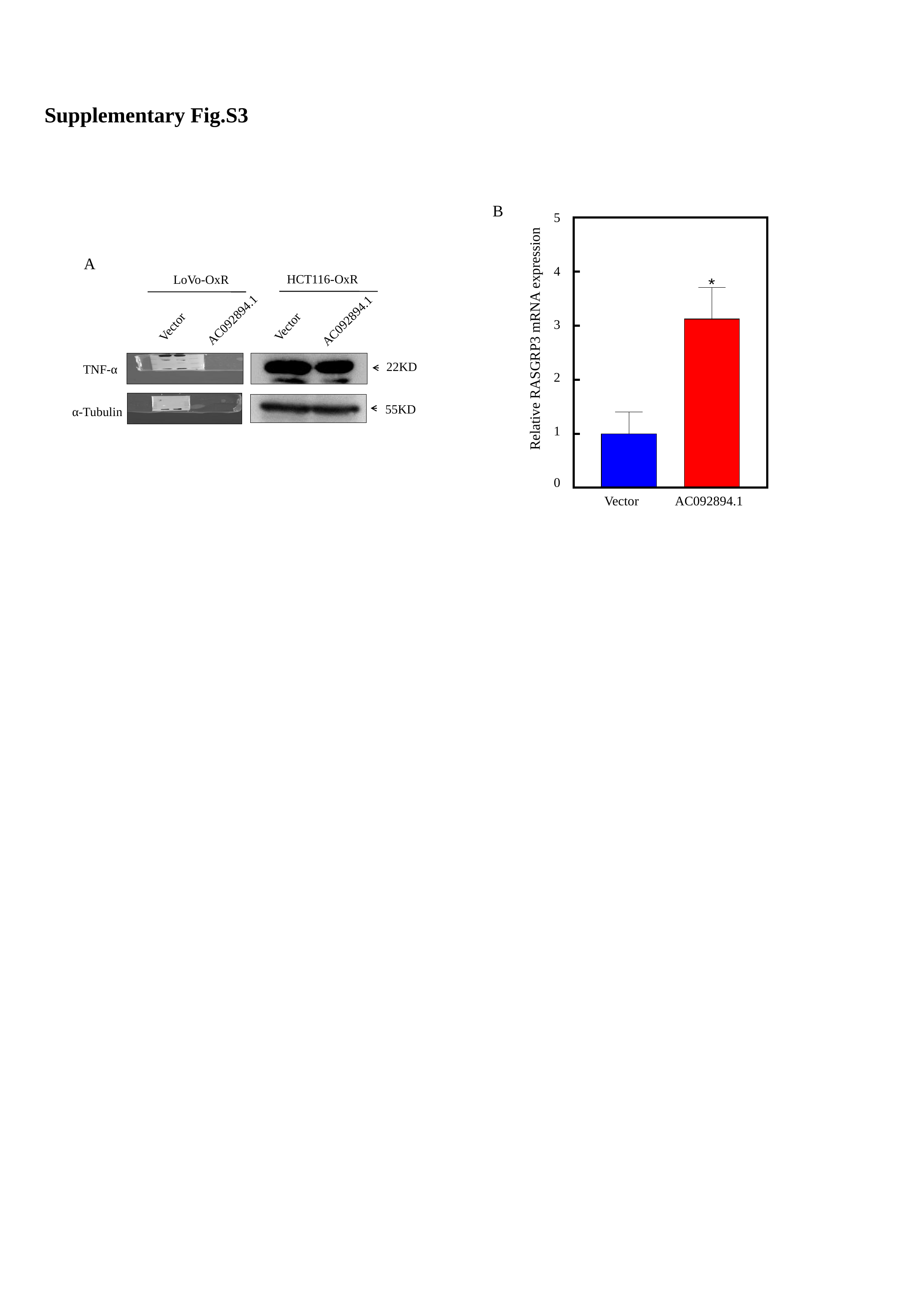

Supplementary Fig.S3
5
4
3
Relative RASGRP3 mRNA expression
2
1
0
Vector
AC092894.1
B
A
HCT116-OxR
LoVo-OxR
AC092894.1
AC092894.1
Vector
Vector
22KD
TNF-α
55KD
α-Tubulin

## Slide 4
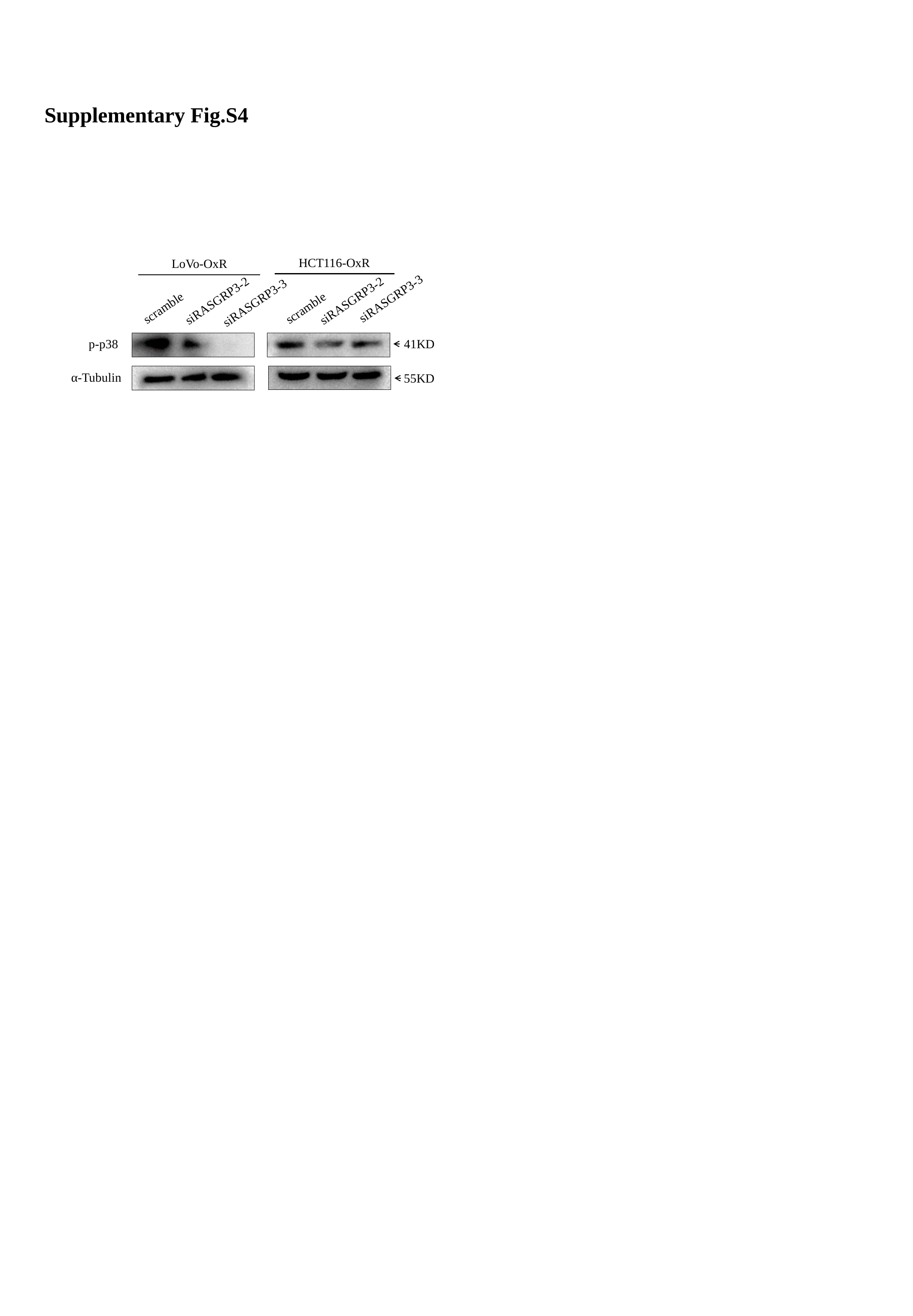

Supplementary Fig.S4
HCT116-OxR
 LoVo-OxR
siRASGRP3-3
siRASGRP3-2
siRASGRP3-2
siRASGRP3-3
scramble
scramble
p-p38
41KD
55KD
α-Tubulin

## Slide 5
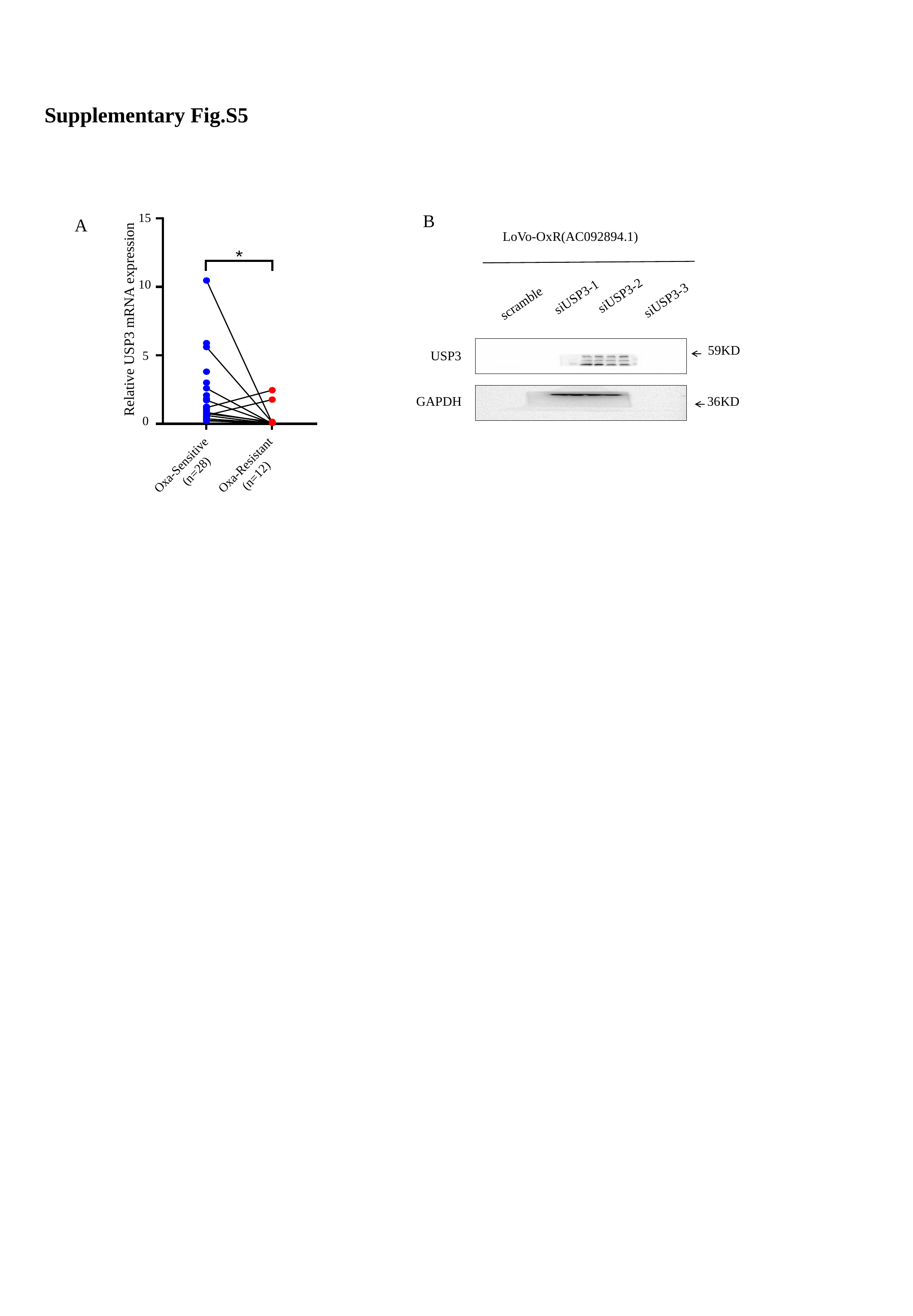

Supplementary Fig.S5
15
10
5
0
Relative USP3 mRNA expression
Oxa-Sensitive
 (n=28)
Oxa-Resistant
(n=12)
B
A
LoVo-OxR(AC092894.1)
siUSP3-2
siUSP3-1
siUSP3-3
scramble
59KD
USP3
GAPDH
36KD

## Slide 6
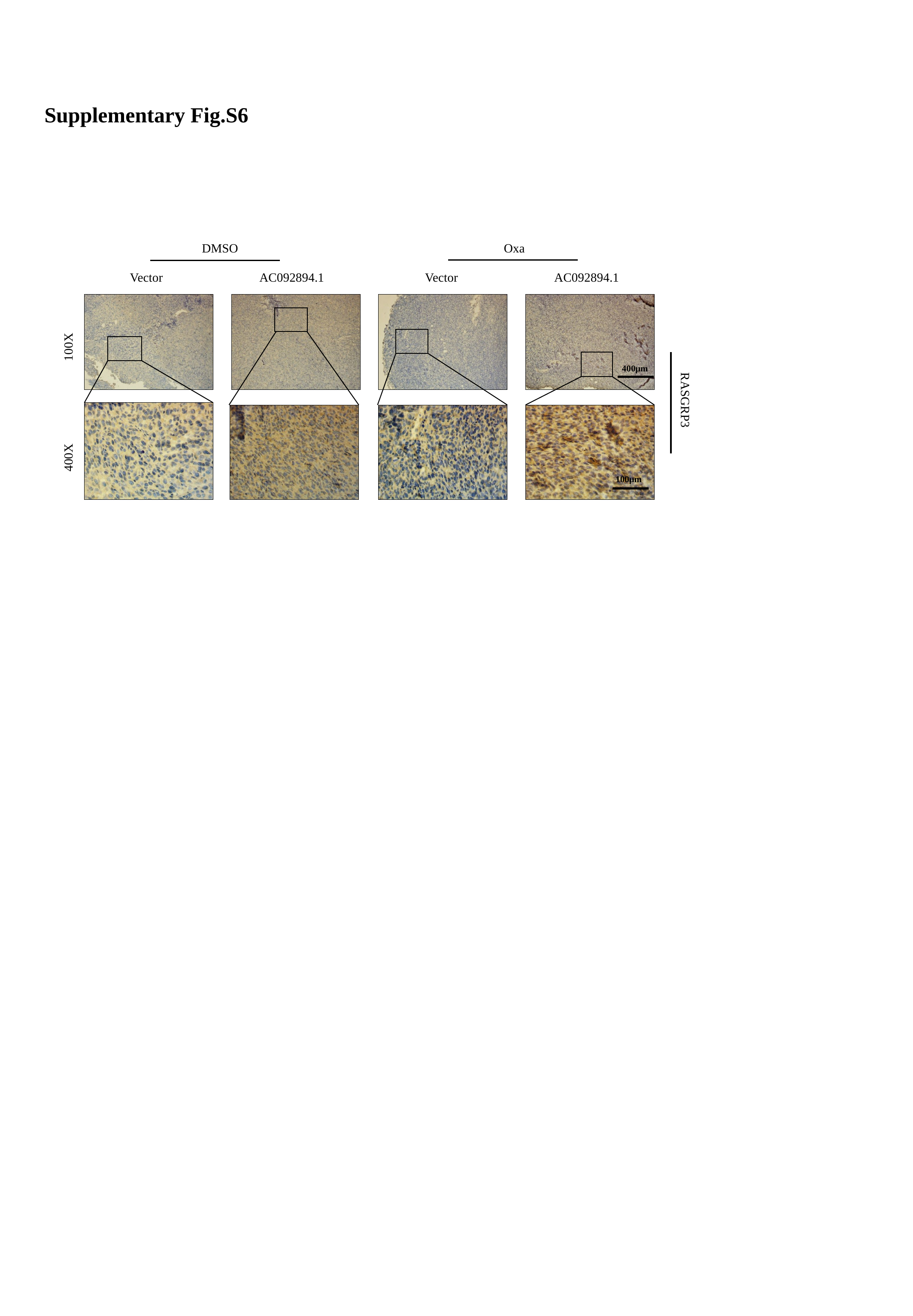

Supplementary Fig.S6
DMSO
Oxa
Vector
AC092894.1
Vector
AC092894.1
100X
RASGRP3
400X
400μm
100μm

## Slide 7
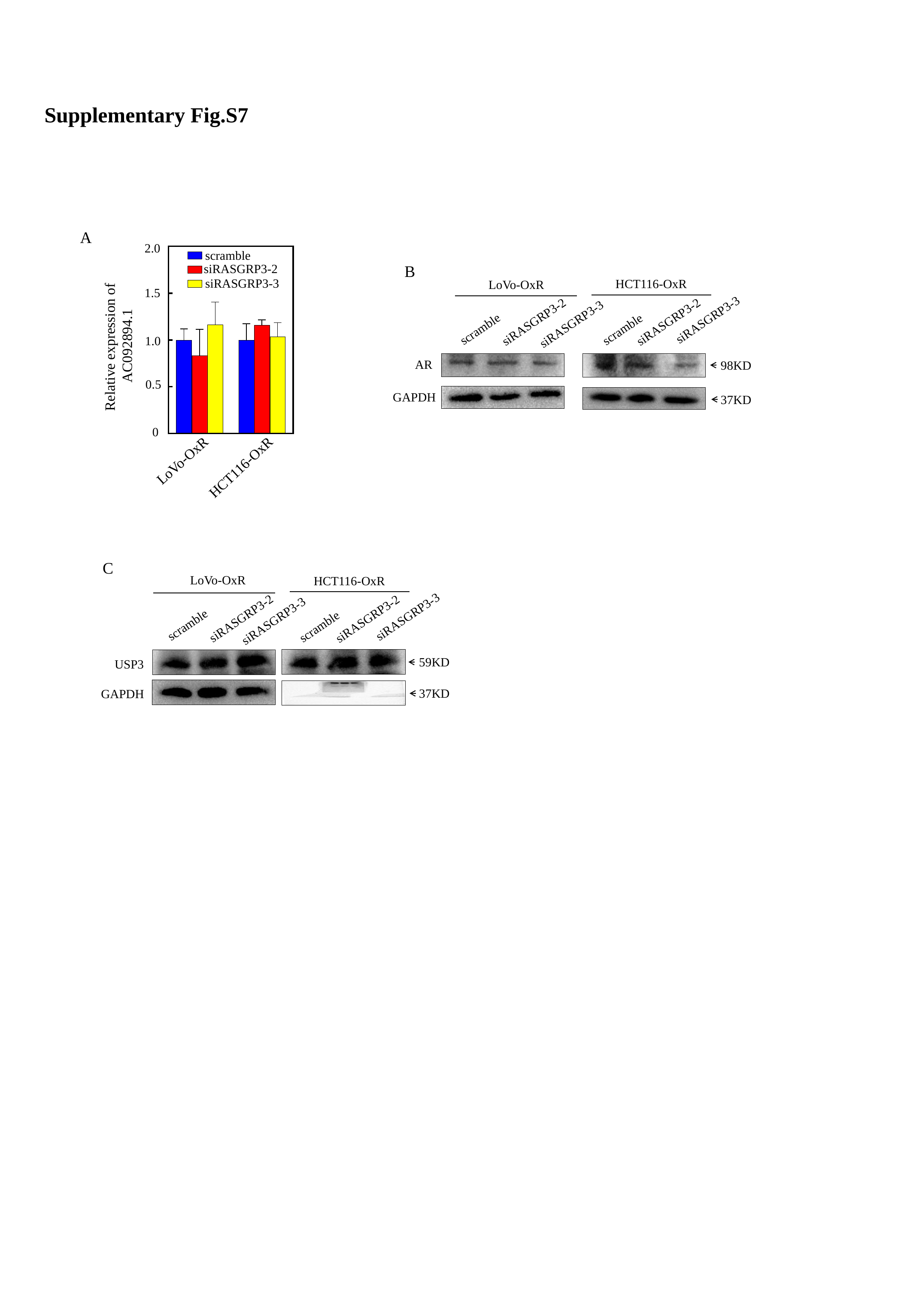

Supplementary Fig.S7
A
2.0
scramble
siRASGRP3-2
siRASGRP3-3
1.5
Relative expression of
 AC092894.1
1.0
0.5
0
LoVo-OxR
HCT116-OxR
B
HCT116-OxR
 LoVo-OxR
siRASGRP3-3
siRASGRP3-2
siRASGRP3-2
siRASGRP3-3
scramble
scramble
AR
98KD
37KD
GAPDH
C
 LoVo-OxR
HCT116-OxR
siRASGRP3-3
siRASGRP3-2
siRASGRP3-2
siRASGRP3-3
scramble
scramble
59KD
USP3
37KD
GAPDH
