# Supplementary material for: Downregulation of AC092894.1 promotes oxaliplatin resistance in colorectal cancer via the USP3/AR/RASGRP3 axis
Source: BMC Med. 2023 Apr 3;21:132. doi: 10.1186/s12916-023-02826-6 (PMC10071743; doi:10.1186/s12916-023-02826-6)
Supplement: Supplementary file 2 — Additional file 2: Table S1. The clinical information of CRC patients. Table S2. The list of qPCR primers in this study. Table S3. Sequence of shRNA. Table S4. List of genes with >2-fold differential expression in the MAPK signalling pathway from RNA-seq results. [file 12916_2023_2826_MOESM2_ESM.docx]

**Table S1** The clinical information of CRC patients.

| case（NO#） | gender | age | Oxa response | case（NO#） | gender | age | Oxa response |
| --- | --- | --- | --- | --- | --- | --- | --- |
| 1# | female | 46 | resistant | 21# | male | 68 | sensitive |
| 2# | female | 58 | resistant | 22# | male | 57 | sensitive |
| 3# | male | 55 | resistant | 23# | male | 70 | sensitive |
| 4# | male | 62 | resistant | 24# | female | 64 | sensitive |
| 5# | male | 64 | resistant | 25# | male | 37 | sensitive |
| 6# | female | 56 | resistant | 26# | male | 72 | sensitive |
| 7# | female | 67 | resistant | 27# | male | 66 | sensitive |
| 8# | male | 28 | resistant | 28# | female | 62 | sensitive |
| 9# | female | 68 | resistant | 29# | male | 43 | sensitive |
| 10# | male | 60 | resistant | 30# | male | 71 | sensitive |
| 11# | male | 69 | resistant | 31# | male | 43 | sensitive |
| 12# | male | 53 | resistant | 32# | male | 58 | sensitive |
| 13# | female | 63 | sensitive | 33# | male | 71 | sensitive |
| 14# | male | 55 | sensitive | 34# | male | 54 | sensitive |
| 15# | male | 64 | sensitive | 35# | male | 54 | sensitive |
| 16# | female | 56 | sensitive | 36# | male | 69 | sensitive |
| 17# | male | 49 | sensitive | 37# | female | 80 | sensitive |
| 18# | male | 58 | sensitive | 38# | male | 66 | sensitive |
| 19# | male | 62 | sensitive | 39# | male | 51 | sensitive |
| 20# | female | 59 | sensitive | 40# | male | 55 | sensitive |

**Table S2** The list of qPCR primers in this study.

|  | Forward Primer | Reverse Primer |
| --- | --- | --- |
| AC092894.1 | 5’-TGCTGAAACTGCCTCCACAA-3’ | 5’-CCCAGGAGCAAGCCAAAGTA-3’ |
| LINC00704 | 5’-ACAGGCAACAGGACTCTGAC-3’ | 5’-GTACAGCCTGCTCGGAGAAC-3’ |
| CACNG4 | 5’-TCTGGTCTGTGGCGGGTGTG-3’ | 5’-GCTGTCGTGGTCGTAGTCATTGTC-3’ |
| EFNA2 | 5’-TTCTCCCTGGGCTTCGAGTT-3’ | 5’-TACAGGGTCTCGTTGGTCGG-3’ |
| EFNA4 | 5’-ACTACTACATCTCGGTGCCC-3’ | 5’-CTGATGTGCCACTCTCTCCA-3’ |
| RASGRP2 | 5’-GCTCAAGTGAACAGCACGTC-3’ | 5’-ACCTTCCCGGAGTCATCGAA-3’ |
| ATF2 | 5’-ATGTGAATTCTGCCAGGCAATAC-3’ | 5’-GGGGTCTGATCAGCCACAAT-3’ |
| ELK1 | 5’-ATCTGTGACGCTGTGGCAGT-3’ | 5’-CCAGCTTGAATTCACCACCA-3’ |
| ELK4 | 5’-TCACGAGCAGTGATCCAAGC-3’ | 5’-GTCAGTATGATGGGTGTCTGTG-3’ |
| JUND | 5’-CCCCTTCGGTTCTTTCGACC-3’ | 5’-CGGGCGAACCAAGGATTACA-3’ |
| DDIT3 | 5’-CTGGAACCTGAGGAGAGAGTGTT-3’ | 5’-CATCTGCTTTCAGGTGTGGTG-3’ |
| RASGRP3 | 5’-TGATCAACCTGCTCACGCTT-3’ | 5’-GGGAGGTAGGCTGCGATTTA-3’ |
| NTRK2 | 5’-GGAATTGGGTTGGAGCAGGA-3’ | 5’-CACCTCGAGGCCGGG-3’ |
| RAC2 | 5’-TGGCCAAGGAGATTGACTCGG-3’ | 5’-GCCTCGTCGAACACGGTTT-3’ |
| IL1A | 5’-GCGTTTGAGTCAGCAAAGAAGT-3’ | 5’-CAGAGACAGATGATCAATGGAGGA-3’ |
| AR | 5’-CTACATCAAGGAACTCGATCGT-3’ | 5’-CATGTGTGACTTGATTAGCAGG-3’ |
| GAPDH | 5′-GACTCATGACC ACAGTCCATGC-3’ | 5′-CAGGTCAGGTCCACCACTGA-3′ |

**Table S3** Sequence of shRNA

| Name | Sequence |
| --- | --- |
| shNC | CCGGGGTTCTCCGAACGTGTCACGTCTCGAGACGTGACACGTTCGGAGAACCTTTTTG |
| shAC092894.1-1 | TTCTCTATTATTATTTTACTTAT |
| shAC092894.1-2 | GGCTAATTTTTTGTATTTTTAGT |
| shAC092894.1-3 | AGCATAGTTTGGTTTTATACATT |
| shAC092894.1-4 | GGCAGATAAGAGAAAAATGATTG |

**Table S4** List of genes with >2-fold differential expression in the MAPK signalling pathway from RNA-seq results.

| Gene ID | Gene Name | Pathway Name |
| --- | --- | --- |
| ENSG00000075461 | CACNG4 | MAPK signaling pathway |
| ENSG00000099617 | EFNA2 | MAPK signaling pathway |
| ENSG00000243364 | EFNA4 | MAPK signaling pathway |
| ENSG00000068831 | RASGRP2 | MAPK signaling pathway |
| ENSG00000126767 | ELK1 | MAPK signaling pathway |
| ENSG00000130522 | JUND | MAPK signaling pathway |
| ENSG00000115966 | ATF2 | MAPK signaling pathway |
| ENSG00000158711 | ELK4 | MAPK signaling pathway |
| ENSG00000175197 | DDIT3 | MAPK signaling pathway |
| ENSG00000115008 | IL1A | MAPK signaling pathway |
| ENSG00000128340 | RAC2 | MAPK signaling pathway |
| ENSG00000148053 | NTRK2 | MAPK signaling pathway |
| ENSG00000152689 | RASGRP3 | MAPK signaling pathway |
